# Pro-Apoptotic Activity of Artichoke Leaf Extracts in Human HT-29 and RKO Colon Cancer Cells

**DOI:** 10.3390/ijerph18084166

**Published:** 2021-04-15

**Authors:** Milena Villarini, Mattia Acito, Raffaella di Vito, Samuele Vannini, Luca Dominici, Cristina Fatigoni, Rita Pagiotti, Massimo Moretti

**Affiliations:** 1 Unit of Public Health, Department of Pharmaceutical Sciences, University of Perugia, via del Giochetto, 06122 Perugia, Italy; milena.villarini@unipg.it (M.V.); mattia.acito@studenti.unipg.it (M.A.); raffaella.divito@studenti.unipg.it (R.d.V.); samuele.vannini@hotmail.com (S.V.); dominici.luca@virgilio.it (L.D.); cristina.fatigoni@unipg.it (C.F.); 2 Unit of Plant Biology, Department of Pharmaceutical Sciences, University of Perugia, via del Giochetto, 06122 Perugia, Italy; rita.pagiotti@unipg.it

**Keywords:** *Cynara scolymus*, artichoke, HT-29 and RKO tumour cells, antitumour activity, cell proliferation, comet assay, apoptosis

## Abstract

(1) Background: *Cynara cardunculus* L. subsp. *scolymus* (L.) Hegi, popularly known as artichoke, is an herbaceous plant belonging to the Asteraceae family. Artichoke leaf extracts (ALEs) have been widely used in traditional medicine because of their hepatoprotective, cholagogic, hypoglycaemic, hypolipemic and antibacterial properties. ALEs are also recognized for their antioxidative and anti-inflammatory activities. In this study, we evaluated the cytotoxic, genotoxic, and apoptotic activities, as well as effect on cell growth of ALEs on human colon cancer HT-29 and RKO cells. HT-29 and RKO cells exhibit a different p53 status: RKO cells express the wild-type protein, whereas HT-29 cells express a p53-R273H contact mutant. (2) Methods: Four different ALEs were obtained by sequential extraction of dried artichoke leaves; ALEs were characterized for their content in chlorogenic acid, cynaropicrin, and caffeoylquinic acids. HT-29 and RKO cells were used for in vitro testing (i.e., cytotoxicity and genotoxicity assessment, cell cycle analysis, apoptosis induction). (3) Results: Two out of the four tested ALEs showed marked effects on cell vitality toward HT-29 and RKO tumour cells. The effect was accompanied by a genotoxic activity exerted at a non-cytotoxic concentrations, by a significant perturbation of cell cycle (i.e., with increase of cells in the sub-G_1_ phase), and by the induction of apoptosis. (4) Conclusions: ALEs rich in cynaropicrin, caffeoylquinic acids, and chlorogenic acid showed to be capable of affecting HT-29 and RKO colon cancer cells by inducing favourable biological effects: cell cycle perturbation, activation of mitochondrial dependent pathway of apoptosis, and the induction of genotoxic effects probably mediated by the induction of apoptosis. Taken together, these results weigh in favour of a potential cancer chemotherapeutic activity of ALEs.

## 1. Introduction

*Cynara cardunculus* L. subsp. *scolymus* (L.) Hegi, popularly known as artichoke, is an herbaceous plant belonging to the Asteraceae family. Artichoke is native to the Mediterranean Basin and it is widely consumed as part of a traditional Mediterranean diet [1]. The immature composite inflorescence (composed of tender inner leaves) and the receptacle represent the edible part of artichoke and regardless of its origin *Cynara scolymus* is widely cultivated in the regions of subtropical climates because of its nutritional benefits and medicinal properties [2]. Artichoke leaf extracts (ALEs) have been widely used in traditional medicine because of their hepatoprotective, cholagogic, hypoglycaemic, hypolipemic and antibacterial properties [3]. ALEs are also recognized for their antioxidative and anti-inflammatory activities [3].

The current growing interest for artichoke is related to the presence, in different parts of the plant, of bioactive components, such as polyphenols (e.g., chlorogenic acid, cynarin, luteolin 7-O-rutinoside, luteolin 7-O-glucoside, and 1,5-di-O-caffeoylquinic acid), inulin, vitamin C, vitamin K and some minerals such as calcium, iron, and zinc [3,4,5].

Cancer is a leading cause of death worldwide, with estimated 18.1 million new cancer cases and 9.6 million cancer deaths in 2018 [6]. In 2018, in Europe, there were an estimated 3.91 million new cases of cancer and 1.93 million deaths from cancer [7]. Colon and rectum cancers are the fourth and the eighth most incident cancer in the world, respectively [6,8]. Taking the data together, colorectal cancer is the third most commonly diagnosed form of cancer globally, comprising 11% of all cancer diagnoses [6,8]. The leading behavioural and dietary (i.e., modifiable) risk factors associated to colorectal cancer are high body mass index, lack of physical activity, low fruit and vegetable intake, diabetes, and alcohol and/or tobacco use [8].

Cancer chemoprevention is defined as “pharmacological intervention with synthetic or naturally occurring compounds that may prevent, inhibit, or reverse carcinogenesis, or prevent the development of invasive cancer” [9]. Both in smokers and no smokers, chemoprevention may be a promising strategy to block, inhibit, reverse, or delay the process of carcinogenesis. Botanical supplements may be a convenient method of administering potentially chemopreventive agents. Some authors found in the artichoke leaf extract many compounds—first of all polyphenols—that could contribute to the prevention and management of some types of cancer. Recent in vitro and in vivo studies demonstrated the ability of artichoke to inhibit the angiogenesis and/or the proliferation of some cancer cell lines. Artichoke extracts affect cell viability of human leukemic K562 cell line [10], and inhibit proliferation and induce apoptotic pathway in human colorectal cancer DLD1 cells [11]. Moreover, cynaropicrin reduces the viability and promotes cytotoxic effects in anaplastic thyroid cancer cells [12], polyphenolic compounds from the edible part of artichoke reduce cell viability, inhibit cell growth, and show inhibitory properties against the invasive breast cancer cell line MDA-MB231 [13]. Studies have also shown that *Cynara scolymus* leaf extracts reduce the growth of mesothelioma xenografted tumours [14].

In this study, we evaluated the cytotoxic, genotoxic, and apoptotic activities, as well as effect on cell growth of ALEs on two different human colon cancer cell lines (i.e., HT-29 and RKO). HT-29 are well-differentiated colorectal adenocarcinoma cells in which ultrastructural features include microvilli [15], whereas RKO are poorly differentiated colon carcinoma cells [16]. Moreover, among other differences, HT-29 and RKO cells exhibit a different p53 status: RKO cells express the wild-type protein, whereas HT-29 cells express a p53-R273H contact mutant [17] (i.e., a p53 having a mutation in residues directly involved in DNA binding).

Considering that artichoke leaves are consumed as food and are used as tea infusions, in this study we have tested four different ALEs obtained by sequential extraction of dried artichoke leaves.

## 2. Materials and Methods

### 2.1. Chemicals and Reagents

Dulbecco’s Modified Eagle Medium (DMEM), foetal bovine serum (FBS), trypsin-EDTA, L-glutamine, antibiotics (penicillin and streptomycin) were purchased from Euroclone SpA (Milan, Italy). Lactate dehydrogenase (LDH) cytotoxicity detection kit was purchased from Takara Bio Inc. (Kyoto, Japan). Hydrochloric acid (HCl), dimethyl sulfoxide (DMSO), ethanol, ethylenediaminetetracetic acid disodium (Na_2_EDTA) and tetrasodium (Na_4_EDTA) salt, sodium chloride (NaCl), and sodium hydroxide (NaOH) were purchased from Carlo Erba Reagenti Srl (Milan, Italy). Dulbecco’s phosphate-buffered saline, pH 7.4 (PBS), ethidium bromide, low- and normal-melting-point agarose (LMPA and NMPA, respectively), 4-nitroquinoline *N*-oxide (4NQO), tris(hydroxymethyl)aminomethane (Tris base), Triton X-100, staurosporine, and valinomycin were obtained from Sigma-Aldrich Srl (Milan, Italy). Acridine orange (AO), 6,4′-diamidino-2-phenylindole (DAPI), 5,5′,6,6′-tetrachloro-1,1′,3,3′-tetraethyl-benzimidazole-carbocyanine iodide (JC-1), Via1-Cassette, and NC-Slide A8 were purchased from ChemoMetec A/S (Allerød, Denmark). Conventional microscope slides and coverslips were supplied by Knittel-Glaser GmbH (Braunschweig, Germany). Distilled water was used throughout the experiments.

### 2.2. Artchoke Leaf Extracts (ALEs) Preparation

The edible part (i.e., head) and leaves of fresh artichoke (*Cynara scolymus* L.) were used for ALEs preparation. Artichoke leaf freeze-dried extract and partially purified mixtures of polyphenolic and terpene compounds were obtained according to a patented procedure (WO 2016/083993 A1).

Briefly, Extract A (ABO-1) was obtained by hydroalcoholic extraction of dried leaves and flower-heads cut into small pieces. Extractions were performed in two sequential steps using ethanol and distilled water. Artichoke dried leaves were firstly extracted with ethanol, the residue was subsequently subjected to a further extraction with distilled water. The collected alcoholic and aqueous solutions were mixed and subjected to precipitation and centrifugation, the precipitate obtained after supernatant removal was collected, freeze dried, and titrated. To obtain the Extract B (ABO-AR-2013-507), ethanol in the supernatant of the hydroalcoholic fraction was removed by a vacuum rotary evaporator, the obtained aqueous solution was subjected to precipitation and centrifugation, and the precipitate obtained after supernatant removal was collected, freeze dried, and titrated. Extract C (ABO-AR-2013-508) corresponds to the insoluble residue of the aqueous supernatant that was obtained by filtration, freeze drying, and titration. Alternatively, to obtain the Extract D (ABO-AR-2013-510), an aliquot of the insoluble filtrate was subjected to adsorption on high-porosity adsorbing resin, the adsorbed fraction was then recovered, concentrated, freeze dried, and titrated.

The highest soluble concentration of the test compounds for in vitro testing was determined according to the protocol for solubility determination proposed by the US National Toxicology Program Interagency Center for the Evaluation of Alternative Toxicological Methods (NICEATM) [18]. The solvents for dissolving ALEs, in the order of preference, were culture medium, DMSO, and ethanol. Briefly, the compounds were initially dissolved in culture medium at a concentration of 20 mg/mL, and a sequence of mixing methods (i.e., vortexing, sonicating, and heating at 37 °C) were followed. If necessary, the volume of solvent was increased, and mixing method repeated, to solubilize the extract at lower concentrations. If the extract was insoluble in the culture medium, the procedure was repeated in DMSO, and then in EtOH. Solubility was considered to be achieved when, upon visual observation, the solution was clear and showed no signs of cloudiness or precipitation [18].

### 2.3. Chemical Characterization of ALEs

The procedures adopted for chemical characterization of ALEs are reported in detail elsewhere (Aboca’s patent WO 2016/083993 A1). Briefly, chlorogenic acid (CA) and cynaropicrin were determined by UHPLC analysis; caffeoylquinic acids (CQAs) were determined spectrophotometrically.

### 2.4. Cell Culture Conditions

In vitro testing was performed on human colon cancer HT-29 and RKO cell lines. The cells were maintained in a humidified incubator with 5% CO_2_ at 37 °C and routinely cultured in the DMEM supplemented with 10% heat inactivated FBS, 100 IU/mL penicillin, and 100 µg/mL streptomycin. Cells were maintained as a monolayer in 75 cm^2^ flasks until confluency, and were sub-cultured by dispersal with 0.05% trypsin/0.02% EDTA, for a contact time of 5 min at 37 °C.

For cytotoxicity testing, the colonocytes (2.5 × 10^4^ cells/well) were seeded on 96-well culture plates with 100 µL medium (1% FBS). After 18 h, the cells were treated for 4, 8, 16 and 24 h with 9 scalar concentrations of the four test samples. Extract solubility and vehicle toxicity were the limiting factors to define the top test concentrations. The negative control was DMEM or 1% DMSO. For all other assays (i.e., cell proliferation, apoptosis, cell cycle, and genotoxicity testing), HT-29 and RKO cells were sub-cultured for 24 h in 6-well plates at 10^6^ cells per well, the cells were then exposed to the three highest non-cytotoxic concentrations of each test sample. For genotoxicity testing, following the guidelines of the Comet Assay Expert Panel indicating an optimal exposure length of 3 to 6 h [19], HT-29 and RKO cells were incubated with test samples for 4 h. For all other assays, the cells were treated for 4, 8, 16 and 24 h. At the end of the treatments, cells were detached with trypsin/EDTA, resuspended with complete medium, and analyzed for genotoxicity, proliferation, apoptosis, and cell cycle. For each treatment protocol, negative (i.e., solvent of test compound) and positive controls were included. All treatments were carried out in triplicate.

### 2.5. Cytotoxicity Testing: LDH Leakage Assay

Cytotoxicity testing is the first, mandatory step before performing tests evaluating other cellular parameters (e.g., genotoxicity) [20]. We assessed the possible cytotoxicity of test samples by evaluating 9 scalar concentrations in the LDH (lactate dehydrogenase) leakage assay [21]. Cells were treated as described above. At the end of the treatments, the cell culture media were collected for the detection of LDH activity using a colorimetric kit (i.e., Takara’s LDH Cytotoxicity Detection Kit) according to the manufacturer’s protocol. Absorbance at 492 nm was recorded using a Tecan Sunrise microplate reader (Tecan Italia Srl, Milan, Italy). Cytotoxicity was expressed as percent LDH activity present in supernatants of extract-treated cells relative to that in control cells.

### 2.6. Cell Count and Vitality: Acridine Orange/DAPI Double Staining

The number of total and viable cells was estimated by staining cell populations with acridine orange (AO) and DAPI fluorophores. After cells treatment, aliquots of cell suspensions were loaded into Via1-Cassette. The inside of the Via1-Cassette is coated with AO (staining the entire population of cells) and DAPI (staining the non-viable cells). The Via1-Cassettes were then placed in a NucleoCounter^®^ NC-3000™ (Chemometec, Allerød, Denmark) cytometer where cell concentration and viability were determined. Total cell concentration in Via1-Cassette was displayed on a personal computer using the NucleoView software. Viability of treated cells was calculated as a percent of control.

### 2.7. Genotoxicity Testing: Comet Assay

Genotoxicity was investigated by applying the single-cell microgel-electrophoresis (comet) assay performed according to the original three-layer protocol [19], with minor modifications [21]. In addition, 4-NQO (1 µM) was used as positive control. Briefly, immediately after the in vitro treatment, the cells harvested by trypsinization were centrifuged at 70× *g* for 8 min, and pellets were gently resuspended in 300 µL of 0.7% LMPA maintained at 37 °C, 60 µL aliquots were immediately spread onto microscope slides precoated with 1.0% NMPA. After brief agarose solidification at 4 °C, the cell-containing microgels were covered with a top layer (75 µL) of 0.7% LMPA. After solidification of the agarose, the slides were immersed in an ice-cold lysis solution (10 mM Tris-HCl, 2.5 M NaCl, 100 mM Na_2_EDTA, and 1% Triton X-100; pH 10) and left to stand for 18 h at 4 °C. After removal from the lysis solution, the slides were placed horizontally in an electrophoresis chamber and left for 20 min at 4 °C in the high-pH electrophoresis buffer (300 mM NaOH, 1 mM Na_4_EDTA, pH > 13) to allow the DNA to unwind. The electrophoresis was carried out on ice in the same buffer for 20 min at 1.0 V/cm and 300 mA. After electrophoresis, the slides were removed and washed twice with the neutralization buffer (0.4 M Tris-HCl, pH 7.5). Finally, the cells were fixed in 70% ethanol for 5 min, dried at room temperature, and stored at least overnight before microscopic observation. All the above-described operations were performed under low lighting conditions to avoid additional DNA damage.

Slides were stained immediately before analysis using 50 µL of ethidium bromide (10 µg/mL). For each experimental point, duplicate slides were prepared. The extent of DNA damage was evaluated quantitatively by an epi-fluorescent microscope (Olympus BX41, Tokyo, Japan) under a 100 W high-pressure mercury lamp (HSH-1030-L, Ushio, Japan) at 200× magnification. Comet images were taken live with a high-sensitivity black and white charge-coupled device (CCD) camera (PE2020, Pulnix Europe Ltd., Basingstoke, UK). Tail intensity (TI, percent of fluorescence migrated in the comet tail) was evaluated by using a computerized image analysis system (Comet Assay III, Perceptive Instruments, Suffolk, UK). Fifty comets per slide were analyzed. The percentage of hedgehog cells (cells with TI of around 85–90% and above) was reported and hedgehog comets were included in the analysis of genotoxicity [22].

### 2.8. Cell Cycle Analysis of Fixed Cells: DAPI Assay

The effects of the tested samples on cell cycle distribution in HT-29 and RKO cells were analyzed by staining cells with DAPI (in stoichiometric relationship to DNA content). After treatment, the cells were harvested by trypsinization and pooled with cells floating in the medium; the cells were then centrifuged, and the pellets were fixed with 4.5 mL of 70% cold ethanol for at least 2 h. The cells were then centrifuged for 5 min at 500× *g* and washed with PBS. The pellets were stained with 1 µg/mL DAPI, incubated for 5 min at 37 °C, and finally loaded into NC-Slide A8 [23]. Cell cycle profiles were carried out using the fixed cell cycle-DAPI assay protocol in a NucleoCounter NC-3000 fluorescence image cytometer. In a given population, cells will be distributed among three major phases of the cell cycle: G_0_/G_1_ phase (one set of paired chromosomes per cell), S phase (DNA synthesis with variable amount of DNA), and G_2_/M phase (two sets of paired chromosomes per cell, prior to cell division); hypodiploid (sub-G_1_) cells were also taken into consideration during cell cycle analysis. DNA content histograms were used to demarcate cells in different phases of the cell cycle.

### 2.9. Early Apoptosis Analysis: Mitochondrial Membrane Potential Assay (ΔΨm)

HT-29 and RKO cells were cultured as above. Loss of mitochondrial membrane potential (ΔΨm), a distinctive feature of early stages of apoptosis, was assessed by using the dual-emission potential-sensitive probe JC-1. In early apoptotic cells, where the mitochondrial membrane potential collapses, the monomeric JC-1 remains cytosolic and stains the cytosol with a green colour. On the other hand, in non-apoptotic cells, JC-1 impulsively forms complexes with intense red fluorescence [24,25]. In brief, after treatment, approximately 1 × 10^6^ cells were centrifuged, and the pellets were incubated for 15 min at 37 °C with 12.5 µL of JC-1 (200 µg/mL). After that, the cells were centrifuged, washed twice with PBS, resuspended in 0.25 mL of DAPI (1 µg/mL in PBS), and analyzed immediately. Mitochondrial membrane depolarization was revealed as a decrease in the red/green fluorescence intensity ratio. NucleoCounter NC-3000 automated system was used for data analysis. Valinomycin 0.5 µM was used as positive control.

### 2.10. Late Apoptosis Analysis: DNA Fragmentation Assay

This late event of apoptosis was detected by DNA content analysis to discriminate cells having less than 2 C DNA (DNA content of a diploid somatic nucleus), so-called sub-G_1_ cells. In fact, during apoptosis calcium- and magnesium-dependent nucleases are activated to degrade DNA. The consequence of nicks and double-strand breaks formed along the DNA molecule is DNA fragmentation [24,26,27]. For the test, the cells were first permeabilized with ethanol—during this procedure the low-molecular-weight DNA inside the apoptotic cells leaks out and is removed from the sample during a subsequent washing step—and the high-molecular-weight DNA retained in the cells was then stained with DAPI; this fluorochrome binds only to double-stranded DNA, and the intensity of DAPI will correspond to the amount of DNA (thus the G_0_/G_1_ peak will have half the amount of fluorescent intensity of the G_2_ peak). DAPI stained cells were loaded into NC-Slide A8 and cellular fluorescence was quantified by a NucleoCounter NC-3000 automated image analysis system. Staurosporine (1 μM) was used as positive control.

### 2.11. Statistical Analysis

After testing the normal distribution of data with the Kolmogorov-Smirnov test, the results were summarized as the mean ± standard error of mean (SEM) of at least three separate experiments. Comparisons among groups were performed by one-way analysis of variance (ANOVA) followed by Dunnett’s post hoc analysis for pair-wise comparisons between ALEs and untreated (control) cells. Pearson’s correlation coefficients (*r*) were calculated to investigate concentration−response relationships. The level of significance was set at *p* < 0.05 for all statistical analyses. The statistical package SPSS (SPSS Inc., Chicago, IL, USA) was used for statistical analysis.

## 3. Results

### 3.1. ALEs Main Components and Solubility

Results of phytochemical analysis of ALEs, and top concentrations for in vitro cytotoxicity testing are summarized in Table 1.

### 3.2. Cytotoxicity

For cytotoxicity testing, HT-29 and RKO cells were exposed for 4, 8, 16 and 24 h to ALEs over a range of concentrations. Cytotoxicity testing and subsequent experiments in RKO cells were performed only with the two ALEs which showed relevant results in HT-29 cells (i.e., Extracts A and D). Starting by the top concentration (see Table 1), eight two-fold serial dilutions were carried out to obtain nine scalar concentrations for each extract. ALEs did not show any detectable cytotoxic effect on HT-29 and RKO cells (data not shown).

The results of LDH leakage assay determined the choice of concentrations to be evaluated afterwards. The next steps were then conducted using the three highest concentrations which did not show cytotoxic effects.

### 3.3. Cell Count and Vitality

**Figure 1:**
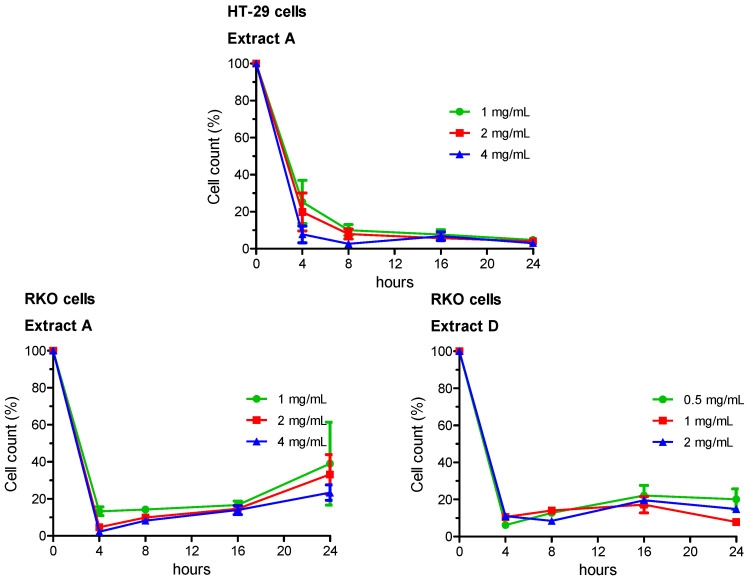
Effects of scalar concentrations of artichoke leaf Extracts A and D on HT-29 and RKO cells vitality. Experimental groups comprised cells treated for 4, 8, 16 and 24 h. Percent of growth expressed as percent of negative control (taken as unit, 100%). The results of each experimental set are summarized as the mean (±SEM) of three independent experiments. Statistically significant differences are highlighted in Supplementary Table S1.

Growing capabilities (i.e., vitality) of viable cells were evaluated by a double-staining technique using orange acridine and DAPI fluorochromes. Experimental groups comprised cells treated for 4, 8, 16 and 24 h. Live/dead cell double staining has been utilized to detect and count viable and total HT-29 or RKO cells. Results were then expressed as percentage variation of cell vitality with respect to the negative control, taken as unit (100%). The whole set of data is reported in Supplementary Tables S1 (HT-29 cells) and S2 (RKO cells), the most relevant results are summarized in Figure 1.

In HT-29 cells, vitality decreased in a concentration- and time-dependent manner following treatment with the Extract A. Minimal or no effects were observed for Extracts B and C, which did not noticeably influence HT-29 cell growth at any concentration and any time of treatment, with respect to the negative control. Sample D showed an intermediate pattern, with a statistically significant reduced vitality in several experimental sets.

In RKO cells, vitality decreased in a concentration- and time-dependent manner following exposure of cells to both Extracts A and D.

### 3.4. Comet Assay

Genotoxicity testing was conducted using the three highest concentrations which did not show cytotoxic effects in LDH leakage assay. The top concentration, as evaluated in test method protocol for solubility determination, was always included. Table 2 summarizes the results of the comet assay.

Exposure of HT-29 cells to Extracts A and D increased the extent of DNA damage for all the three tested concentrations, as compared to negative/solvent control. On the contrary, treatments with Extracts B and C did not result in any increased level of primary DNA damage. Moreover, for Extracts A and D, a significant increased frequency of hedgehog cells was also observed in HT-29 cells. Results of linear correlation analysis showed that for Extracts A and D tail intensity tended to rise with the test sample concentration (Extract A, *r* = 0.846, *p* = 0.001; Extract D, *r* = 0.662, *p* = 0.019). Furthermore, in HT-29 cells, Extract A also showed a significant concentration-effect relationship between extract concentration and percentage of hedgehog cells (*r* = 0.766, *p* = 0.004).

Analogously, when Extracts A and D were tested in RKO cells, exposure of cells to ALEs increased the extent of DNA damage at all the three tested concentrations. Furthermore, a significant increased frequency of hedgehog cells was also observed.

### 3.5. Cell Cycle Analysis

**Figure 2:**
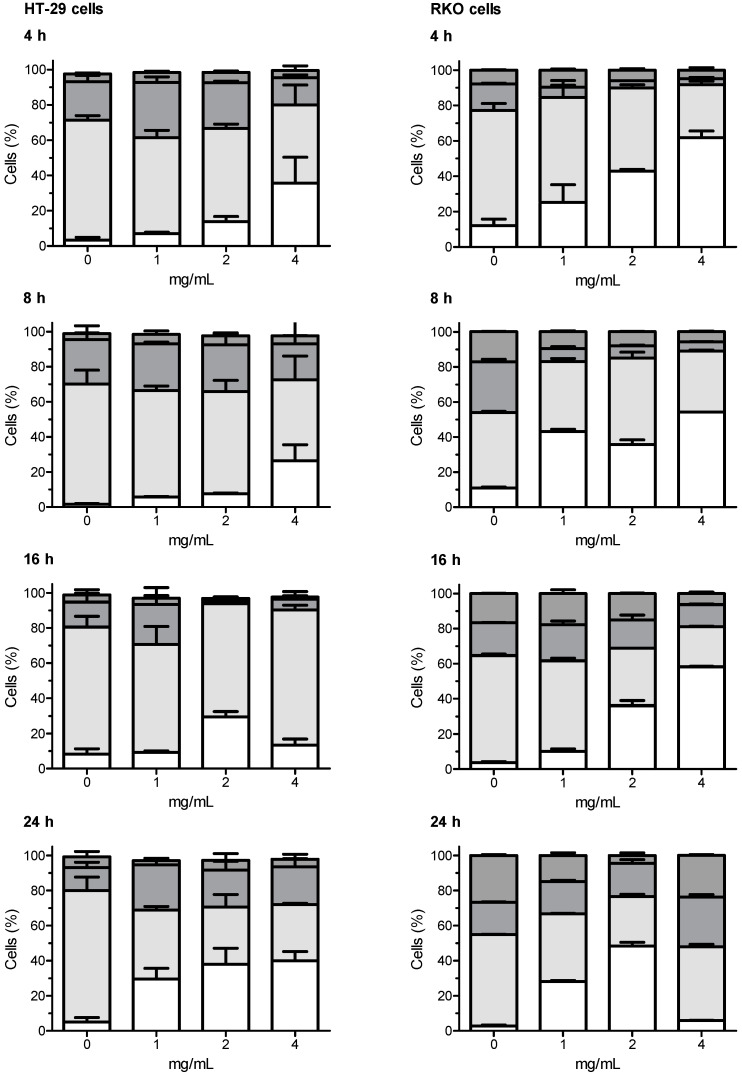
Effects of artichoke leaf Extract A on HT-29 and RKO cell cycle determined using the three highest concentrations which did not show cytotoxic effects in the LDH leakage test, starting by the top concentration (see Table 1). Legend: 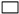 Sub-G_1_, 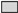 G_0_/G_1_, 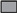 S, 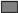 G_2_/M cells, respectively.

The whole set of data regarding the distribution of treated and control cells in different phases of cell cycle is presented in supplementary Tables S3 (HT-29 cells) and S4 (RKO cells); the most relevant results (i.e., Extract A) are summarized in Figure 2.

All the tested extracts have shown capabilities of interfering with the cell cycle of HT-29 and RKO cells. In both cell lines, the most marked effects have been observed with the artichoke leaf Extract A. After incubation of HT-29 cells with Extract A, a concentration-dependent increased proportion of cells in the sub-G_1_ phase was generally observed, the increase in sub-G_1_ population was accompanied by a significant decrease of cells in the G_0_/G_1_ phase, compared with untreated control cells. A similar trend as for HT-29 cells was also observed when RKO cells were challenged with Extract A. In this case, the concentration dependent increased proportion of cells in the sub-G_1_ phase associated with concomitant decreased proportion of cells in the G_0_/G_1_ phase was even more marked.

### 3.6. Early and Late Apoptosis

**Figure 3:**
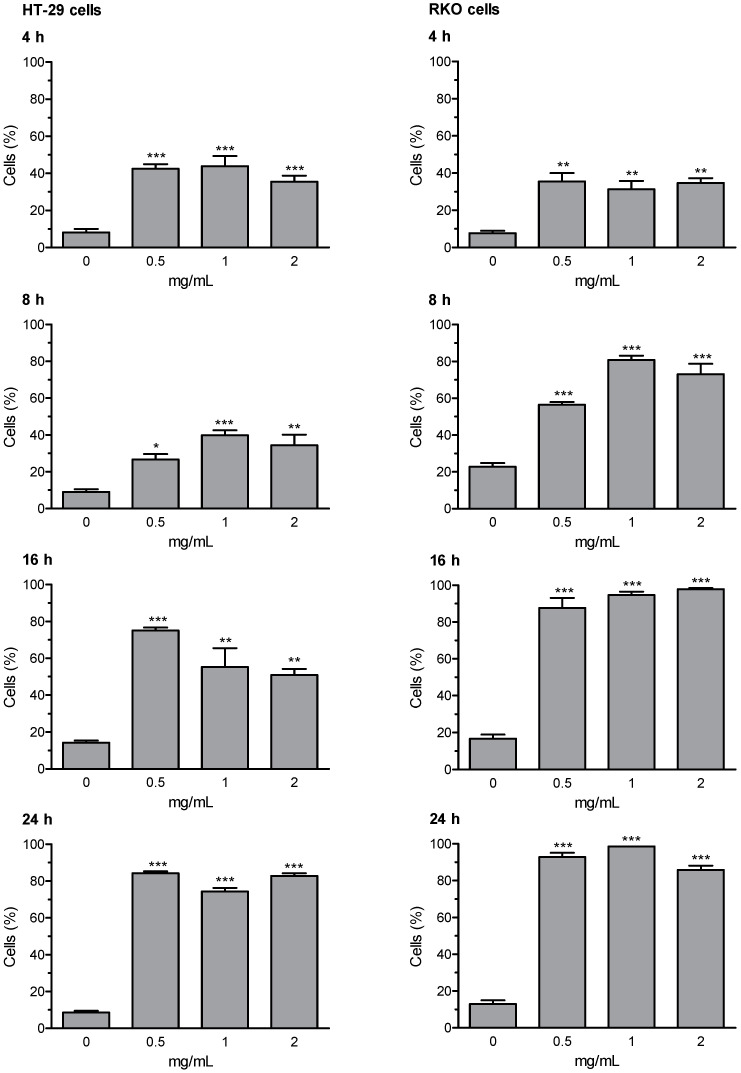
Effects of artichoke leaf Extract D on early apoptosis (mitochondrial membrane depolarization; ΔΨm) induction in HT-29 and RKO cells. Experimental groups comprised cells treated for 4, 8, 16 and 24 h with the three highest concentrations which did not show cytotoxic effects in the LDH leakage test, starting by the top concentration (see Table 1). Results are expressed as percent of apoptotic cells; experimental sets are summarized as the mean (± SEM) of three independent experiments. Statistical analysis: * (*p* < 0.05), ** (*p* < 0.01), or *** (*p* < 0.001) indicate significantly differences, as compared with control cells, one-way ANOVA followed by Dunnett’s post hoc analysis.

**Figure 4:**
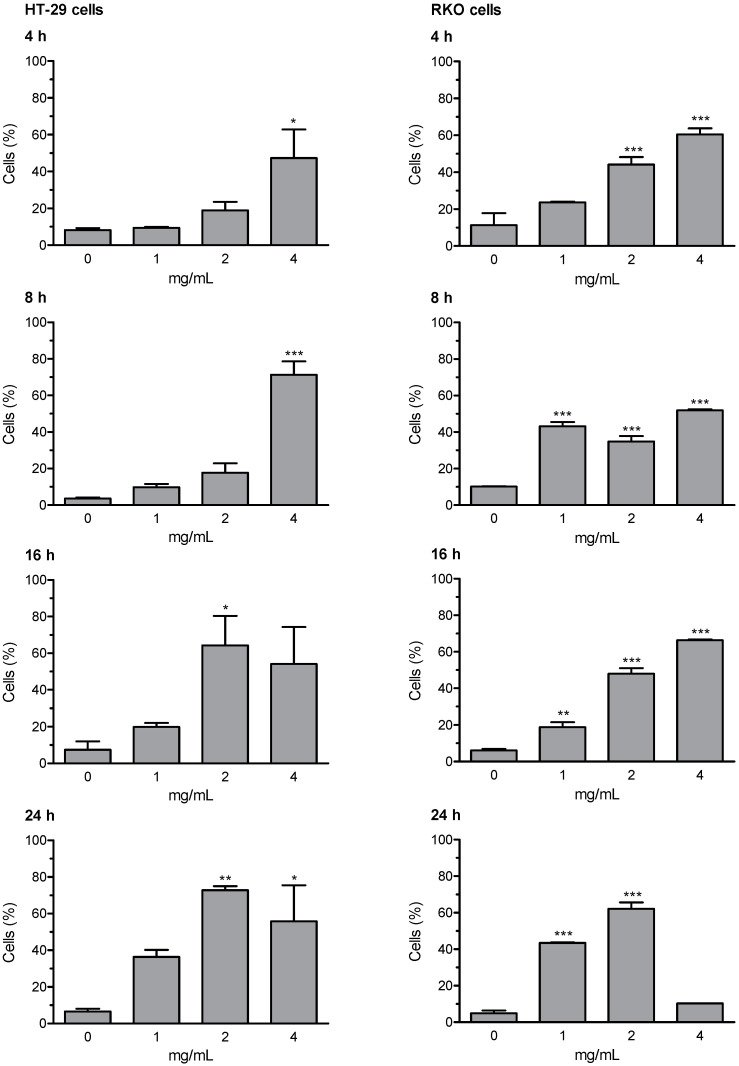
Effects of artichoke leaf Extract A on late apoptosis (DNA fragmentation) induction in HT-29 and RKO cells. Experimental groups comprised cells treated for 4, 8, 16 and 24 h with the three highest concentrations which did not show cytotoxic effects in the LDH leakage test, starting by the top concentration (see Table 1). Results are expressed as percent of apoptotic cells; experimental sets are summarized as the mean (±SEM) of three independent experiments. Statistical analysis: * (*p* < 0.05), ** (*p* < 0.01), or *** (*p* < 0.001) indicate significantly differences, as compared with control cells, one-way ANOVA followed by Dunnett’s post hoc analysis.

Loss of mitochondrial membrane potential (ΔΨm) and the induction of DNA fragmentation were investigated using the fluorescence image cytometer NucleoCounter NC-3000. Early and late apoptosis results are expressed as percentage of apoptotic cells; valinomycin (0.5 µM) was used as a positive control for mitochondrial membrane depolarization; staurosporine (1 μM) was used as positive control for DNA fragmentation. The whole set of data for early and late apoptosis is presented in supplementary Tables S5 (HT-29 cells) and S6 (RKO cells); the most relevant results (i.e., Extract D for early apoptosis, and Extract A for late apoptosis) are summarized in Figure 3 and Figure 4. The results of representative experiments illustrating the shift in fluorescence of JC-1 in RKO cells treated with the artichoke leaf Extract A relative to negative and positive control are shown in Supplementary Figure S1.

Extracts B and C were not able to activate the apoptotic program. Whereas, Extracts A and D were effective in inducing apoptosis, in both HT-29 and RKO cells. Extract D induced an increase of cells in early and late apoptosis as early as 4 h of treatment. After prolongation of incubation time up to 8, 16, and 24 h the percentages of depolarized cells and cells with DNA fragmentation were further increased in both cell lines, with more marked effects showed in RKO cells. Apoptosis was induced by Extract A similarly in both cell lines, with significantly increased percentage of cells showing high rated of DNA fragmentation (i.e., late apoptotic cells). The highest effect in terms of induction of late apoptosis (more than 70% cells in late apoptosis stages) was observed in both cell lines after 16- and 24-h treatment with Extract A.

## 4. Discussion

Recently, functional foods (like herbs, grains and spices) have gained interest as chemo-preventive agents because of their considerable amounts of bioactive compounds that can contribute to prevent or reverse the multi-step processes of carcinogenesis [9,28]. For this goal, many plant extracts and plant chemicals have been investigated in recent times. Recently, it has been shown that extracts and bioactive molecules from artichoke (i.e., apigenin, luteolin, cynaropicrin) possess a high antioxidant capacity [29,30], are effective in supporting the treatment of dyslipidaemia [31,32,33], and have some significant antitumour activity [3].

In in vitro assays, purified compounds or fruit/vegetable extracts are used more often than of crude or lyophilised plant. However, the analysis of isolated constituents does not consider the interferences of food matrix components or a closely relation between food matrix and the bioavailability of bioactive compounds [34,35]. In this study, we have investigated whether phytocomplexes in 4 different ALEs (i.e., as obtained by sequential extraction of dried artichoke leaves) are capable of affecting colon cancer cells, represented in the present in vitro approach by HT-29 and RKO tumour cells.

Our results demonstrated that the tested ALEs did not have any remarkable cytotoxicity on HT-29 cells, even at high concentrations. However, HT-29 and RKO colon cancer cells treated with Extracts A and D showed significantly diminished growing capabilities. Moreover, Extracts A and D strongly induced DNA damage in HT-29 and RKO colonocytes and increased the percentage of hedgehog cells, which can be produced during apoptosis. Furthermore, cell cycle of HT-29 and RKO cells was affected by treatment with these extracts, showing a significant increase in sub-G_1_ cells. Conversely, Extracts B and C did not interfere with cell growth and did not significantly induce loss of mitochondrial potential and DNA fragmentation.

Overall, by considering the results obtained for DNA damage, apoptosis, and cell cycle, Extracts A and D could be supposed to be capable of exerting in vitro antitumour effects in HT-29 and RKO colon cancer cells. Many anticancer drugs induce apoptosis through molecular mechanisms mediated by mitochondrial dysfunction [36]. In this work, we demonstrated that exposure of HT-29 and RKO tumour cells to artichoke leaf Extracts A and D leads to the dysfunction of mitochondria, and the observed genotoxic effects are likely produced through the induction of apoptosis.

Our results are in agreement with antiproliferative effects reported by other authors for ALEs and/or purified phytochemicals extracted from artichokes [11,14,37,38,39,40]. In addition, as in this study, ALEs and/or artichoke phytochemicals have been reported to induce apoptosis of tumour cells in in vitro approaches through mitochondrial/caspase dependent pathways [13,14,37,38,40,41,42].

In the present in vitro approach, we evaluated the cytotoxic, genotoxic, and apoptotic activities, as well as effect on cell growth of ALEs on two different human colon cancer cell lines (i.e., HT-29 and RKO). Among other differences [15,16], HT-29 and RKO cells exhibit a different p53 status: RKO cells express the wild-type protein, whereas HT-29 cells express a p53-R273H contact mutant [17] (i.e., a p53 having a mutation in residues directly involved in DNA binding). Mutations in the human p53 gene are the most frequent genetic event occurring during carcinogenesis in a wide variety of tumours [43]; in cell lines, loss of p53 activity is usually associated with several specific markers, such as defects in cell-cycle arrest or apoptosis after DNA damage [44]. RKO (p53 wildtype) cells demonstrated to have a higher sensitivity to ALEs, compared with HT-29 (p53 mutated) cells. Cell vitality was significantly affected in HT-29 cells by only artichoke leaf Extract A, whereas both Extracts A and D were capable of affecting cell vitality in RKO cells. Anyway, both extracts significantly interfered with cell cycle and induced apoptosis in both cell lines, including HT-29 cells.

## 5. Conclusions

Among the four tested ALEs obtained by sequential extraction of dried artichoke leaves, in this in vitro approach the most interesting results have been obtained with Extracts A and D. These extracts were capable of affecting colon cancer cells (i.e., HT-29 and RKO cells) by inducing favourable biological effects, such as: cell cycle perturbation along with increase of cells in sub-G_1_ phase, combined with the induction of DNA fragmentation and activation of mitochondrial dependent pathway of apoptosis, and the induction of genotoxic effects probably mediated by the induction of apoptosis. Taken together, these results weigh in favour of a potential cancer chemotherapeutic activity of ALEs.

In conclusion, we can summarize that edible part of artichoke would be an important natural source of beneficial chemical products against to colorectal cancer, and it has interesting potentialities in development of related anticancer drugs and/or combined therapies.

## 6. Patents

Artichoke (*Cynara scolymus* L.) leaf extracts were obtained according to an Aboca’s patented procedure (WO 2016/083993 A1).

## Figures and Tables

**Table 1:** Main components and solubility of tested artichoke leaf extracts (ALEs).

ALEs	Cynaropicrin	CQAs ^1^	CA ^1^	Solubility ^2^
Extract A	2.05%	11.85%	5.40%	4 mg/mL **^3^**
Extract B	0.44%	1.96%	0.08%	20 µg/mL **^4^**
Extract C	0.24%	2.48%	0.44%	20 µg/mL **^4^**
Extract D	3.91%	30.00%	12.73%	2 mg/mL **^3^**

^1^ CQAs, total caffeoylquinic acids; CA, chlorogenic acid. ^2^ Based on the US National Toxicology Program Interagency Center for the Evaluation of Alternative Toxicological Methods (NICEATM) test method protocol for solubility determination [18]. ^3,4^ Soluble in the culture medium or dimethyl sulfoxide (DMSO), respectively.

**Table 2:** Primary DNA damage in HT-29 and RKO cells exposed for 4 h to different concentrations of ALEs. Extent of DNA strand breakage is expressed in terms of tail intensity (% DNA migrated in the comet tail); results of each experimental set are summarized as the mean value of three independent experiments (±SEM).

	HT-29		RKO	
ALEs	Tail Intensity (% DNA)	Hedgehog Cells(%)	Tail Intensity (% DNA)	Hedgehog Cells (%)
Extract A ^1^				
1 mg/mL	45.65 ± 6.37 *	16.67 ± 2.40	50.45 ± 10.17 *	10.5 ± 3.5
2 mg/mL	85.23 ± 1.88 *	80.00 ± 4.62 *	84.39 ± 3.05 *	51.0 ± 2.0 *
4 mg/mL	84.03 ± 2.47 *	69.33 ± 16.71 *	83.38 ± 1.06 *	47.0 ± 3.0 *
Extract B ^1^				
5 µg/mL	0.93 ± 0.11	0.00 ± 0.00		
10 µg/mL	1.48 ± 0.44	0.67 ± 0.67		
20 µg/mL	1.54 ± 0.36	0.00 ± 0.00		
Extract C ^1^				
5 µg/mL	1.98 ± 0.27	0.00 ± 0.00		
10 µg/mL	1.55 ± 0.41	1.33 ± 0.67		
20 µg/mL	0.73 ± 0.10	0.00 ± 0.00		
Extract D ^1^				
0.5 mg/mL	86.50 ± 4.50 *	74.00 ± 21.01 *	90.19 ± 2.34 *	66.0 ± 3.0 *
1 mg/mL	90.16 ± 1.65 *	78.00 ± 8.00 *	87.28 ± 0.52 *	60.0 ± 4.0 *
2 mg/mL	85.51 ± 6.50 *	57.33 ± 25.98	68.93 ± 7.85 *	20.5 ± 6.5
Controls:				
DMEM ^2^	0.38 ± 0.16	0.00 ± 0.00	1.11 ± 0.57	0.00 ± 0.00
DMSO ^3^	0.86 ± 0.43	0.00 ± 0.00		
4NQO ^4^	14.52 ± 3.58	0.67 ± 0.67	37.80 ± 2.14	0.00 ± 0.00

^1^ Primary DNA damage was determined using the three highest concentrations which did not show cytotoxic effects in the lactate dehydrogenase (LDH) leakage test, starting by the top concentration (see Table 1). ^2,3^ Negative control for Samples A, D, and B, C, respectively. ^4^ Positive control. Statistical analysis: * indicates significantly higher levels of primary DNA damage or number of hedgehog cells, as compared with negative control (*p* < 0.05), one-way ANOVA followed by Dunnett’s post hoc analysis.

## Data Availability

All data generated or analysed during this study are included in this published article (and its Supplementary Figures and Tables).

## Supplementary Materials

The following are available online at https://www.mdpi.com/article/10.3390/ijerph18084166/s1, Table S1: effects of various ALEs concentrations on HT-29 cells vitality. Experimental groups comprised cells treated for 4, 8, 16 and 24 h. Percent of growth is expressed as percent of negative control (taken as unit, 100%). Results of each experimental set are summarized as the mean (±SEM) of three independent experiments. Table S2: effects of various ALEs concentrations on RKO cells vitality. Experimental groups comprised cells treated for 4, 8, 16 and 24 h. Percent of growth is expressed as percent of negative control (taken as unit, 100%). Results of each experimental set are summarized as the mean (±SEM) of three independent experiments. Table S3: cell cycle analysis^1^ in HT-29 cells exposed to different concentrations of artichoke leaf Extract A. Results are expressed as percentage of cells in the different cell cycle phases; data are summarized as the mean (±SEM) of three independent experiments. Table S4: cell cycle analysis in RKO cells exposed to different concentration of artichoke leaf Extract A. Results are expressed as a percentage of cells in the different cell cycle phases; data are summarized as the mean (±SEM) of three independent experiments. Table S5: effects of various ALEs concentrations on early (mitochondrial membrane depolarization; ΔΨm) and late (DNA fragmentation) apoptosis induction in HT-29 cells. Experimental groups comprised cells treated for 4, 8, 16 and 24 h. Results are expressed as percent of apoptotic cells, experimental sets are summarized as the mean (±SEM) of three independent experiments. Table S6: effects of various ALEs concentrations on early (mitochondrial membrane depolarization; ΔΨm) and late (DNA fragmentation) apoptosis induction in RKO cells. Experimental groups comprised cells treated for 4, 8, 16 and 24 h. Results are expressed as percent of apoptotic cells, experimental sets are summarized as the mean (± SEM) of three independent experiments. Figure S1: determination of apoptosis (early events) by evaluation of mitochondrial membrane potential (ΔΨm). Green and red fluorescence of the viable cells displayed in scatterplots: [*A*] negative control; [*B*] positive control (0.5 µM valinomycin); [*C*], [*D*] artichoke leaf Extracts A (1 mg/mL), and D (0.5 mg/mL), respectively.

## References

[1] Rondanelli M., Giacosa A., Morazzoni P., Guido D., Grassi M., Morandi G., Bologna C., Riva A., Allegrini P., Perna S. (2016). MediterrAsian Diet Products That Could Raise HDL-Cholesterol: A Systematic Review. BioMed Res. Int..

[2] Lattanzio V., Kroon P.A., Linsalata V., Cardinali A. (2009). Globe Artichoke: A Functional Food and Source of Nutraceutical Ingredients. J. Funct. Foods.

[3] Ben Salem M., Affes H., Ksouda K., Dhouibi R., Sahnoun Z., Hammami S., Zeghal K.M. (2015). Pharmacological Studies of Artichoke Leaf Extract and Their Health Benefits. Plant Foods Hum. Nutr. Dordr. Neth..

[4] Pandino G., Lombardo S., Mauromicale G. (2011). Mineral Profile in Globe Artichoke as Affected by Genotype, Head Part and Environment. J. Sci. Food Agric..

[5] Negro D., Montesano V., Grieco S., Crupi P., Sarli G., De Lisi A., Sonnante G. (2012). Polyphenol Compounds in Artichoke Plant Tissues and Varieties. J. Food Sci..

[6] Bray F., Ferlay J., Soerjomataram I., Siegel R.L., Torre L.A., Jemal A. (2018). Global Cancer Statistics 2018: GLOBOCAN Estimates of Incidence and Mortality Worldwide for 36 Cancers in 185 Countries. CA Cancer J. Clin..

[7] Ferlay J., Colombet M., Soerjomataram I., Dyba T., Randi G., Bettio M., Gavin A., Visser O., Bray F. (2018). Cancer Incidence and Mortality Patterns in Europe: Estimates for 40 Countries and 25 Major Cancers in 2018. Eur. J. Cancer Oxf. Engl..

[8] Rawla P., Sunkara T., Barsouk A. (2019). Epidemiology of Colorectal Cancer: Incidence, Mortality, Survival, and Risk Factors. Gastroenterol. Rev..

[9] Gullett N.P., Ruhul Amin A.R.M., Bayraktar S., Pezzuto J.M., Shin D.M., Khuri F.R., Aggarwal B.B., Surh Y.-J., Kucuk O. (2010). Cancer Prevention with Natural Compounds. Semin. Oncol..

[10] Russo A., Perri M., Cione E., Di Gioia M.L., Nardi M., Cristina Caroleo M. (2017). Biochemical and Chemical Characterization of Cynara Cardunculus L. Extract and Its Potential Use as Co-Adjuvant Therapy of Chronic Myeloid Leukemia. J. Ethnopharmacol..

[11] Simsek E.N., Uysal T. (2013). In Vitro Investigation of Cytotoxic and Apoptotic Effects of Cynara L. Species in Colorectal Cancer Cells. Asian Pac. J. Cancer Prev. APJCP.

[12] Lepore S.M., Maggisano V., Lombardo G.E., Maiuolo J., Mollace V., Bulotta S., Russo D., Celano M. (2019). Antiproliferative Effects of Cynaropicrin on Anaplastic Thyroid Cancer Cells. Endocr. Metab. Immune Disord. Drug Targets.

[13] Mileo A.M., Di Venere D., Linsalata V., Fraioli R., Miccadei S. (2012). Artichoke Polyphenols Induce Apoptosis and Decrease the Invasive Potential of the Human Breast Cancer Cell Line MDA-MB231. J. Cell. Physiol..

[14] Pulito C., Mori F., Sacconi A., Casadei L., Ferraiuolo M., Valerio M.C., Santoro R., Goeman F., Maidecchi A., Mattoli L. (2015). Cynara Scolymus Affects Malignant Pleural Mesothelioma by Promoting Apoptosis and Restraining Invasion. Oncotarget.

[15] Fogh J., Trempe G., Fogh J. (1975). New Human Tumor Cell Lines. Human Tumor Cells In Vitro.

[16] Brattain M.G., Levine A.E., Chakrabarty S., Yeoman L.C., Willson J.K., Long B. (1984). Heterogeneity of Human Colon Carcinoma. Cancer Metastasis Rev..

[17] Berg K.C.G., Eide P.W., Eilertsen I.A., Johannessen B., Bruun J., Danielsen S.A., Bjørnslett M., Meza-Zepeda L.A., Eknæs M., Lind G.E. (2017). Multi-Omics of 34 Colorectal Cancer Cell Lines—a Resource for Biomedical Studies. Mol. Cancer.

[18] NICEATM/ICCVAM (2006). Test Method Protocol for Solubility Determination; In Vitro Cytotoxicity Validation Study Phase III.

[19] Tice R.R., Agurell E., Anderson D., Burlinson B., Hartmann A., Kobayashi H., Miyamae Y., Rojas E., Ryu J.C., Sasaki Y.F. (2000). Single Cell Gel/Comet Assay: Guidelines for in Vitro and in Vivo Genetic Toxicology Testing. Environ. Mol. Mutagen..

[20] Di Nunzio M., Valli V., Tomás-Cobos L., Tomás-Chisbert T., Murgui-Bosch L., Danesi F., Bordoni A. (2017). Is Cytotoxicity a Determinant of the Different in Vitro and in Vivo Effects of Bioactives?. BMC Complement. Altern. Med..

[21] Moretti M., Cossignani L., Messina F., Dominici L., Villarini M., Curini M., Marcotullio M.C. (2013). Antigenotoxic Effect, Composition and Antioxidant Activity of Dendrobium Speciosum. Food Chem..

[22] Koppen G., Azqueta A., Pourrut B., Brunborg G., Collins A.R., Langie S.A.S. (2017). The next Three Decades of the Comet Assay: A Report of the 11th International Comet Assay Workshop. Mutagenesis.

[23] Respondek M., Beberok A., Rok J., Rzepka Z., Wrześniok D., Buszman E. (2018). MIM1, the Mcl-1—Specific BH3 Mimetic Induces Apoptosis in Human U87MG Glioblastoma Cells. Toxicol. In Vitro.

[24] Villarini M., Pagiotti R., Dominici L., Fatigoni C., Vannini S., Levorato S., Moretti M. (2014). Investigation of the Cytotoxic, Genotoxic, and Apoptosis-Inducing Effects of Estragole Isolated from Fennel (Foeniculum Vulgare). J. Nat. Prod..

[25] Salvioli S., Ardizzoni A., Franceschi C., Cossarizza A. (1997). JC-1, but Not DiOC6(3) or Rhodamine 123, Is a Reliable Fluorescent Probe to Assess ΔΨ Changes in Intact Cells: Implications for Studies on Mitochondrial Functionality during Apoptosis. FEBS Lett..

[26] Nagata S. (2000). Apoptotic DNA Fragmentation. Exp. Cell Res..

[27] Nagata S., Nagase H., Kawane K., Mukae N., Fukuyama H. (2003). Degradation of Chromosomal DNA during Apoptosis. Cell Death Differ..

[28] Vlaykova T., Dimitrova I., Pavlov I., Tacheva T. (2013). Cancer Prevention—Dietary Anticarcinogens. Medicine.

[29] Turkiewicz I.P., Wojdyło A., Tkacz K., Nowicka P., Hernández F. (2019). Antidiabetic, Anticholinesterase and Antioxidant Activity vs. Terpenoids and Phenolic Compounds in Selected New Cultivars and Hybrids of Artichoke Cynara Scolymus L.. Mol. Basel Switz..

[30] Salekzamani S., Ebrahimi-Mameghani M., Rezazadeh K. (2019). The Antioxidant Activity of Artichoke (Cynara Scolymus): A Systematic Review and Meta-Analysis of Animal Studies. Phytother. Res. PTR.

[31] Santos H.O., Bueno A.A., Mota J.F. (2018). The Effect of Artichoke on Lipid Profile: A Review of Possible Mechanisms of Action. Pharmacol. Res..

[32] Sahebkar A., Pirro M., Banach M., Mikhailidis D.P., Atkin S.L., Cicero A.F.G. (2018). Lipid-Lowering Activity of Artichoke Extracts: A Systematic Review and Meta-Analysis. Crit. Rev. Food Sci. Nutr..

[33] Wider B., Pittler M.H., Thompson-Coon J., Ernst E. (2013). Artichoke Leaf Extract for Treating Hypercholesterolaemia. Cochrane Database Syst. Rev..

[34] Phan M.A.T., Paterson J., Bucknall M., Arcot J. (2018). Interactions between Phytochemicals from Fruits and Vegetables: Effects on Bioactivities and Bioavailability. Crit. Rev. Food Sci. Nutr..

[35] Speijers G., Bottex B., Dusemund B., Lugasi A., Tóth J., Amberg-Müller J., Galli C.L., Silano V., Rietjens I.M.C.M. (2010). Safety Assessment of Botanicals and Botanical Preparations Used as Ingredients in Food Supplements: Testing an European Food Safety Authority-Tiered Approach. Mol. Nutr. Food Res..

[36] Green D.R., Kroemer G. (2004). The Pathophysiology of Mitochondrial Cell Death. Science.

[37] Miccadei S., Di Venere D., Cardinali A., Romano F., Durazzo A., Foddai M.S., Fraioli R., Mobarhan S., Maiani G. (2008). Antioxidative and Apoptotic Properties of Polyphenolic Extracts from Edible Part of Artichoke (Cynara Scolymus L.) on Cultured Rat Hepatocytes and on Human Hepatoma Cells. Nutr. Cancer.

[38] Nadova S., Miadokova E., Mucaji P., Grancai D., Cipak L. (2008). Growth Inhibitory Effect of Ethyl Acetate-Soluble Fraction of Cynara Cardunculus L. in Leukemia Cells Involves Cell Cycle Arrest, Cytochrome c Release and Activation of Caspases. Phytother. Res. PTR.

[39] Menghini L., Genovese S., Epifano F., Tirillini B., Ferrante C., Leporini L. (2010). Antiproliferative, Protective and Antioxidant Effects of Artichoke, Dandelion, Turmeric and Rosemary Extracts and Their Formulation. Int. J. Immunopathol. Pharmacol..

[40] Hassabou N.F., Farag A.F. (2020). Anticancer Effects Induced by Artichoke Extract in Oral Squamous Carcinoma Cell Lines. J. Egypt. Natl. Cancer Inst..

[41] Mileo A.M., Di Venere D., Abbruzzese C., Miccadei S. (2015). Long Term Exposure to Polyphenols of Artichoke (Cynara Scolymus L.) Exerts Induction of Senescence Driven Growth Arrest in the MDA-MB231 Human Breast Cancer Cell Line. Oxid. Med. Cell. Longev..

[42] Liu T., Zhang J., Han X., Xu J., Wu Y., Fang J. (2019). Promotion of HeLa Cells Apoptosis by Cynaropicrin Involving Inhibition of Thioredoxin Reductase and Induction of Oxidative Stress. Free Radic. Biol. Med..

[43] O’Connor P.M., Jackman J., Jondle D., Bhatia K., Magrath I., Kohn K.W. (1993). Role of the P53 Tumor Suppressor Gene in Cell Cycle Arrest and Radiosensitivity of Burkitt’s Lymphoma Cell Lines. Cancer Res..

[44] Oren M. (2003). Decision Making by P53: Life, Death and Cancer. Cell Death Differ..

