# Supplementary material for: Pro-Apoptotic Activity of Artichoke Leaf Extracts in Human HT-29 and RKO Colon Cancer Cells
Source: Int J Environ Res Public Health. 2021 Apr 15;18(8):4166. doi: 10.3390/ijerph18084166 (PMC8071198; doi:10.3390/ijerph18084166)
Supplement: Supplementary file 1 [file ijerph-18-04166-s001.pdf]

Table S1. Effects of various ALEs concentrations on HT-29 cells vitality. Experimental groups comprised cells treated for 4, 8, 16 and 24 h. Percent of growth is expressed as percent of negative control (taken as unit, 100%). Results of each experimental set are summarized as the mean (± SEM) of three independent experiments.

	**Treatment time**			
**ALEs**	**4 h**	**8 h**	**16 h**	**24 h**
Extract A [1]				
1 mg/mL	25.31 ± 11.61***	10.08 ± 2.94***	7.73 ± 2.60***	4.80 ± 0.67***
2 mg/mL	19.88 ± 10.23***	7.99 ± 2.73***	5.70 ± 1.63***	4.08 ± 1.78***
4 mg/mL	7.75 ± 4.58***	2.67 ± 1.65***	6.78 ± 2.32***	3.03 ± 0.92***
p (r)	*0.004 (-0.764)*	*0.007 (-0.728)*	*0.014 (-0.685)*	*0.012 (-0.694)*
Extract B [1]				
5 µg/mL	138.21 ± 18.99	79.22 ± 16.12	123.81 ± 18.85	99.32 ± 16.16
10 µg/mL	134.08 ± 23.77	94.64 ± 25.84	134.17 ± 23.02	89.26 ± 10.85
20 µg/mL	93.33 ± 2.45	103.58 ± 27.23	149.92 ± 21.76	86.91 ± 13.25
p (r)	*0.528 (-0.202)*	*0.701 (0.124)*	*0.065 (0.548)*	*0.338 (-0.303)*
Extract C [1]				
5 µg/mL	93.87 ± 12.92	120.41 ± 53.90	184.78 ± 58.21	125.03 ± 30.30
10 µg/mL	106.88 ± 27.26	84.30 ± 17.05	235.24 ± 135.19	152.72 ± 96.67
20 µg/mL	104.16 ± 27.35	89.86 ± 31.52	149.40 ± 82.83	114.29 ± 63.27
p (r)	*0.779 (0.091)*	*0.645 (-0.149)*	*0.742 (0.106)*	*0.873 (0.052)*
Extract D [1]				
0.5 mg/mL	78.55 ± 16.91	73.37 ± 4.30	39.38 ± 1.42***	58.14 ± 14.04
1 mg/mL	72.55 ± 12.17	57.63 ± 6.11*	43.48 ± 9.08**	77.20 ± 17.85
2 mg/mL	40.81 ± 5.48*	50.25 ± 13.91*	48.56 ± 8.93**	104.26 ± 38.34
p (r)	*0.002 (-0.803)*	*0.003 (-0.785)*	*0.067 (-0.454)*	*0.613 (0.132)*

[1] Cell count and viability were determined using the three highest concentrations which did not show cytotoxic effects in the LDH leakage test, starting by the top concentration (see Table 1). Statistical analysis: *(p<0.05), **(p<0.01), or ***(p<0.001) indicate significantly different number of cells, as compared with negative control, one-way ANOVA followed by Dunnett's post hoc analysis.

Table S2. Effects of various ALEs concentrations on RKO cells vitality. Experimental groups comprised cells treated for 4, 8, 16 and 24 h. Percent of growth is expressed as percent of negative control (taken as unit, 100%). Results of each experimental set are summarized as the mean (± SEM) of three independent experiments.

	**Treatment time**			
**ALEs**	**4 h**	**8 h**	**16 h**	**24 h**
Extract A [1,2]				
1 mg/mL	25.31 ± 11.61***	10.08 ± 2.94***	7.73 ± 2.60***	4.80 ± 0.67*
2 mg/mL	19.88 ± 10.23***	7.99 ± 2.73***	5.70 ± 1.63***	4.08 ± 1.78*
4 mg/mL	7.75 ± 4.58***	2.67 ± 1.65***	6.78 ± 2.32***	3.03 ± 0.92**
p (r)	*0.004 (-0.764)*	*0.007 (-0.728)*	*0.014 (-0.685)*	*0.012 (-0.694)*
Extract D [1,2]				
0.5 mg/mL	78.55 ± 16.91***	73.37 ± 4.30	39.38 ± 1.42***	58.14 ± 14.04***
1 mg/mL	72.55 ± 12.17***	57.63 ± 6.11***	43.48 ± 9.08***	77.20 ± 17.85***
2 mg/mL	40.81 ± 5.48***	50.25 ± 13.91***	48.56 ± 8.93***	104.26 ± 38.34***
p (r)	*0.002 (-0.803)*	*0.003 (-0.785)*	*0.067 (-0.454)*	*0.613 (0.132)*

[1] Cell count and viability were determined using the three highest concentrations which did not show cytotoxic effects in the LDH leakage test, starting by the top concentration (see Table 1). Statistical analysis: *(p<0.05), **(p<0.01), or ***(p<0.001) indicate significantly different number of cells, as compared with negative control, one-way ANOVA followed by Dunnett's post hoc analysis.

Table S3. Cell cycle analysis[1] in HT-29 cells exposed to different concentrations of artichoke leaf extract A. Results are expressed as % of cells in the different cell cycle phases; data are summarized as the mean (± SEM) of three independent experiments.

ALEs		4 h	8 h	16 h	24 h
Extract A					
1 mg/mL	Sub-G_1	7.04 ± 0.80	5.67 ± 0.35	9.26 ± 0.86	29.52 ± 6.11
	G_0/G_1	54.34 ± 4.20	60.70 ± 2.59	61.36 ± 10.29	39.34 ± 2.04**
	S	31.34 ± 3.14	26.72 ± 0.97	22.80 ± 9.65	25.81 ± 3.73
	G_2/M	5.60 ± 0.76	5.31 ± 2.01	5.55 ± 1.47	2.42 ± 0.59
2 mg/mL	Sub-G_1	13.77 ± 2.96	7.59 ± 0.40	29.48 ± 3.02**	37.95 ± 9.17*
	G_0/G_1	52.88 ± 2.43	58.21 ± 6.43	64.38 ± 3.92	32.65 ± 7.16***
	S	26.00 ± 0.73	26.65 ± 6.90	1.66 ± 0.56	21.06 ± 4.99
	G_2/M	5.74 ± 0.76	5.16 ± 1.24	1.19 ± 0.76	5.54 ± 3.81
4 mg/mL	Sub-G_1	35.57 ± 14.81*	26.29 ± 9.15*	13.36 ± 3.48	40.04 ± 5.16**
	G_0/G_1	44.37 ± 11.32	46.27 ± 13.54	76.87 ± 2.71	31.89 ± 0.72***
	S	15.38 ± 1.69	20.38 ± 13.78	6.27 ± 4.24	21.54 ± 7.24
	G_2/M	4.15 ± 2.57	0.71 ± 0.12	1.09 ± 0.79	1.37 ± 0.41
DMEM [2]	Sub-G_1	3.35 ± 1.58	1.52 ± 0.43	8.27 ± 3.06	5.04 ± 2.53
	G_0/G_1	68.02 ± 2.51	68.57 ± 7.91	72.26 ± 6.11	74.97 ± 7.59
	S	21.69 ± 3.68	25.30 ± 7.87	14.27 ± 4.98	13.06 ± 3.06
	G_2/M	4.50 ± 0.61	3.41 ± 0.50	4.04 ± 2.95	6.13 ± 3.08
Extract B					
5 μg/mL	Sub-G_1	8.12 ± 0.43*	14.57 ± 4.03	13.21 ± 1.62	13.39 ± 0.98
	G_0/G_1	60.39 ± 0.76	53.50 ± 5.63	60.95 ± 5.77	63.24 ± 1.07
	S	11.66 ± 1.18	13.51 ± 0.63	15.65 ± 2.28	14.28 ± 0.61
	G_2/M	9.32 ± 0.71*	7.76 ± 1.98	6.55 ± 0.75	6.79 ± 0.99
10 μg/mL	Sub-G_1	8.73 ± 0.90*	12.93 ± 2.36	10.99 ± 1.08	11.29 ± 0.33
	G_0/G_1	55.64 ± 1.05*	55.09 ± 2.61	64.86 ± 4.5	62.70 ± 0.90
	S	10.98 ± 1.33	13.19 ± 1.84	15.44 ± 3.38	16.10 ± 0.90
	G_2/M	8.14 ± 0.59**	12.05 ± 2.44	7.34 ± 0.44	7.33 ± 0.39
20 μg/mL	Sub-G_1	1.11 ± 1.66***	15.81 ± 3.00	11.28 ± 1.29	16.98 ± 0.03*
	G_0/G_1	53.24 ± 1.71**	53.33 ± 2.41	68.72 ± 0.45	62.85 ± 1.68
	S	8.32 ± 0.86*	10.72 ± 1.23*	9.83 ± 2.02	9.84 ± 3.68
	G_2/M	7.13 ± 0.29**	14.00 ± 2.44	7.39 ± 2.70	9.27 ± 2.61
DMSO [2]	Sub-G_1	3.36 ± 0.21	4.11 ± 1.52	8.47 ± 3.38	7.59 ± 2.64
	G_0/G_1	63.13 ± 1.77	71.44 ± 5.94	70.34 ± 4.90	75.23 ± 9.50
	S	17.75 ± 2.91	16.22 ± 0.20	14.55 ± 1.31	10.25 ± 3.93
	G_2/M	13.85 ± 1.49	6.42 ± 3.57	5.31 ± 2.01	5.83 ± 2.53
Extract C					
5 μg/mL	Sub-G_1	8.88 ± 1.48	12.76 ± 2.78	11.51 ± 1.18***	12.14 ± 1.73
	G_0/G_1	59.79 ± 0.74	62.85 ± 0.64	62.51 ± 1.12***	59.10 ± 0.89*
	S	13.25 ± 1.11	11.99 ± 1.32	17.64 ± 0.12	19.15 ± 1.56
	G_2/M	9.63 ± 1.11	8.62 ± 0.79	7.16 ± 0.55*	7.33 ± 1.45
10 μg/mL	Sub-G_1	8.90 ± 0.31	15.4 ± 5.00	12.53 ± 0.38***	12.40 ± 1.57
	G_0/G_1	63.72 ± 4.05	53.9 ± 5.93	63.49 ± 0.74***	59.19 ± 2.20*
	S	12.36 ± 0.65	17.14 ± 1.56	16.46 ± 1.46	18.92 ± 2.45
	G_2/M	8.39 ± 0.27	8.14 ± 2.98	6.00 ± 0.51	8.08 ± 0.73
20 μg/mL	Sub-G_1	10.16 ± 1.55*	13.58 ± 0.27	13.28 ± 0.66***	13.24 ± 1.12
	G_0/G_1	62.38 ± 1.04	53.46 ± 2.39	63.79 ± 1.71***	65.00 ± 0.82
	S	12.78 ± 1.37	12.88 ± 1.84	14.90 ± 1.34	13.94 ± 0.34
	G_2/M	11.86 ± 1.14	7.36 ± 1.92	6.23 ± 0.47	7.63 ± 0.19
DMSO [2]	Sub-G_1	4.41 ± 0.57	4.08 ± 0.98	3.57 ± 1.22	8.98 ± 2.47
	G_0/G_1	54.19 ± 3.42	64.79 ± 1.67	75.04 ± 1.66	67.66 ± 2.10
	S	12.04 ± 3.99	15.53 ± 0.44	15.77 ± 2.09	14.16 ± 1.48
	G_2/M	11.81 ± 1.09	13.38 ± 1.79	4.39 ± 0.87	7.43 ± 0.82
Extract D					
0.5 mg/mL	Sub-G_1	8.51 ± 4.26	12.53 ± 6.45	35.24 ± 8.33*	33.86 ± 4.94**
	G_0/G_1	50.81 ± 1.76*	47.09 ± 1.73	47.42 ± 10.89	55.84 ± 5.49
	S	7.75 ± 1.80	8.88 ± 0.84	7.71 ± 0.74	7.28 ± 0.43
	G_2/M	3.85 ± 0.11	9.28 ± 0.47	4.06 ± 0.83	1.14 ± 0.33***
1 mg/mL	Sub-G_1	8.97 ± 4.49	5.51 ± 2.80	20.98 ± 4.02	20.99 ± 0.19
	G_0/G_1	42.8 ± 2.37**	56.23 ± 1.77	56.69 ± 4.30	55.50 ± 8.23
	S	5.69 ± 0.73	9.60 ± 2.84	10.45 ± 2.09	17.97 ± 7.75
	G_2/M	5.64 ± 0.81	19.72 ± 0.64	3.24 ± 2.36	2.70 ± 0.39**
2 mg/mL	Sub-G_1	8.23 ± 4.19	6.29 ± 3.21	8.52 ± 1.13	27.74 ± 6.35*

	G_0/G_1	44.72 ± 0.01**	54.71 ± 0.91	74.61 ± 3.25	57.21 ± 2.51
	S	8.63 ± 0.80	9.58 ± 1.09	15.04 ± 1.62	12.74 ± 6.03
	G_2/M	6.77 ± 2.40	21.34 ± 0.21	0.78 ± 0.09	1.18 ± 0.70***
DMEM [2]	Sub-G_1	5.04 ± 1.51	3.30 ± 1.79	6.98 ± 3.90	6.77 ± 2.75
	G_0/G_1	60.30 ± 1.59	68.35 ± 8.38	71.34 ± 2.29	66.04 ± 2.53
	S	18.86 ± 4.67	15.35 ± 3.29	16.12 ± 3.86	14.57 ± 1.82
	G_2/M	9.91 ± 4.39	11.91 ± 4.75	4.44 ± 1.34	11.02 ± 2.07

[1] Cell cycle analysis was performed using the three highest concentrations which did not show cytotoxic effects in the LDH leakage test, starting by the top concentration (see Table 1). [2] Negative control. Statistical analysis: *(p<0.05), **(p<0.01), or ***(p<0.001) indicate significantly different number of cells, as compared with negative control, one-way ANOVA followed by Dunnett's post hoc analysis.

Table S4. Cell cycle analysis in RKO cells exposed to different concentration of artichoke leaf extract A. Results are expressed as % of cells in the different cell cycle phases; data are summarized as the mean (± SEM) of three independent experiments.

**ALEs**		**4 h**	**8 h**	**16 h**	**24 h**
Extract A					
1 mg/mL	Sub-G_1	25.12 ± 10.10	43.17 ± 1.20***	10.14 ± 1.35	28.17 ± 0.59***
	G_0/G_1	59.36 ± 9.66	39.90 ± 1.73	51.54 ± 1.43***	38.67 ± 0.13***
	S	5.86 ± 1.02***	7.38 ± 1.12***	20.49 ± 2.15	18.26 ± 0.79
	G_2/M	9.67 ± 0.57	9.66 ± 0.53***	17.82 ± 2.07	14.90 ± 1.51***
2 mg/mL	Sub-G_1	42.86 ± 1.01*	35.67 ± 2.73***	36.07 ± 2.92***	48.40 ± 2.07***
	G_0/G_1	47.06 ± 1.70	49.35 ± 3.44	32.75 ± 0.02***	28.13 ± 1.33***
	S	4.02 ± 0.05***	6.96 ± 0.59***	16.12 ± 2.74	18.95 ± 2.17
	G_2/M	6.07 ± 0.74	8.16 ± 0.18***	15.07 ± 0.20	4.52 ± 1.43***
4 mg/mL	Sub-G_1	61.79 ± 3.79***	54.19 ± 0.03***	58.23 ± 0.26***	5.94 ± 0.07
	G_0/G_1	29.96 ± 1.95**	34.94 ± 0.49*	22.87 ± 0.23***	42.04 ± 1.37***
	S	3.30 ± 0.77***	5.28 ± 0.05***	12.59 ± 0.35	28.35 ± 1.29**
	G_2/M	4.96 ± 1.40	5.73 ± 0.39***	6.32 ± 0.84***	23.69 ± 0.48
DMEM [2]	Sub-G_1	11.89 ± 3.76	10.54 ± 0.73	3.64 ± 0.48	2.79 ± 0.38
	G_0/G_1	64.98 ± 3.72	42.88 ± 0.59	60.01 ± 0.55	52.17 ± 0.29
	S	15.01 ± 0.43	29.02 ± 1.05	17.98 ± 0.24	18.35 ± 0.27
	G_2/M	7.84 ± 0.27	17.14 ±0.31	15.99 ± 0.19	25.89 ± 0.37
Extract D					
0.5 mg/mL	Sub-G_1	33.46 ± 1.95***	26.89 ± 1.93**	14.81 ± 2.17***	14.91 ± 2.87**
	G_0/G_1	50.00 ± 4.56*	58.14 ± 2.68**	11.70 ± 1.17**	18.87 ± 0.06***
	S	6.14 ± 0.51***	6.84 ± 0.19***	9.55 ± 0.28*	11.44 ± 1.17*
	G_2/M	10.41 ± 2.10	8.25 ± 0.88***	3.75 ± 0.59	2.87 ± 0.49
1 mg/mL	Sub-G_1	24.45 ± 0.93*	12.01 ± 3.05	37.03 ± 2.22***	51.16 ± 0.08
	G_0/G_1	61.86 ± 0.81	72.29 ± 2.62***	48.39 ± 1.10*	47.85 ± 0.20
	S	5.55 ± 1.07***	9.51 ± 2.13***	47.12 ± 5.27*	64.90 ± 3.72**
	G_2/M	8.15 ± 1.18	6.21 ± 1.71***	60.90 ± 0.84	52.02 ± 0.21
2 mg/mL	Sub-G_1	25.84 ± 1.95**	18.14 ± 4.32	24.99 ± 0.65*	31.30 ± 2.55**
	G_0/G_1	61.13 ± 2.04	62.07 ± 3.15**	22.71 ± 2.25	31.32 ± 0.70**
	S	6.31 ± 0.36***	5.68 ± 0.41***	29.33 ± 1.71**	19.56 ± 3.02
	G_2/M	6.82 ± 0.22	14.12 ± 0.76	18.69 ± 0.15	18.47 ± 0.20
DMEM [2]	Sub-G_1	12.11 ± 3.69	10.86 ± 0.65	23.19 ± 3.73	2.64 ± 0.24***
	G_0/G_1	65.09 ± 3.91	43.19 ± 0.61	17.20 ± 2.33	1.96 ± 0.44***
	S	14.94 ± 0.44	28.83 ± 1.54	14.42 ± 3.61	4.12 ± 1.86***
	G_2/M	7.87 ± 0.20	17.25 ±0.20	16.67 ± 0.10	26.65 ± 0.48

[1] Cell cycle analysis was performed using the three highest concentrations which did not show cytotoxic effects in the LDH leakage test, starting by the top concentration (see Table 1). [2] Negative control. Statistical analysis: *(*p*<0.05), **(*p*<0.01), or ***(*p*<0.001) indicate significantly different number of cells, as compared with negative control, one-way ANOVA followed by Dunnett's post hoc analysis.

Table S5. Effects of various ALEs concentrations on early (mitochondrial membrane depolarization; ΔΨm) and late (DNA fragmentation) apoptosis induction in HT-29 cells. Experimental groups comprised cells treated for 4, 8, 16 and 24 h. Results are expressed as percent of apoptotic cells, experimental sets are summarized as the mean (± SEM) of three independent experiments.

	**ΔΨm (Early apoptosis)[1]**				**DNA fragmentation (Late apoptosis)[1]**			
**ALEs**	**4 h**	**8 h**	**16 h**	**24 h**	**4 h**	**8 h**	**16 h**	**24 h**
Extract A								
1 mg/mL	26.19 ± 7.11	12.24 ± 3.02	28.51 ± 9.95	3.81 ± 1.83	9.45 ± 0.51	9.76 ± 1.74	19.89 ± 2.12	36.35 ± 3.87
2 mg/mL	1.59 ± 0.35	3.56 ± 2.20	10.86 ± 2.53	1.47 ± 0.39*	18.90 ± 4.63	17.67 ± 5.21	64.29 ± 16.06*	72.83 ± 2.14**
4 mg/mL	1.75 ± 0.36	2.13 ± 0.91*	4.04 ± 1.74	3.38 ± 1.13*	47.25 ± 15.66*	71.29 ± 7.37***	54.15 ± 20.18	55.78 ± 19.64*
DMEM [2]	12.08 ± 1.27	11.82 ± 1.51	8.08 ± 0.78	9.60 ± 2.01	8.19 ± 1.08	3.54 ± 0.67	7.49 ± 4.51	6.52 ± 1.48
Extract B								
5 µg/mL	9.35 ± 1.93	10.98 ± 1.38	12.51 ± 0.95**	12.09 ± 3.32	9.25 ± 1.17*	18.73 ± 5.37*	16.10 ± 2.03***	15.78 ± 0.70**
10 µg/mL	14.36 ± 1.11	10.22 ± 0.83	14.80 ± 1.20***	9.91 ± 2.73	11.12 ± 0.04**	15.42 ± 1.35	13.44 ± 1.10**	14.67 ± 0.44*
20 µg/mL	14.37 ± 3.77	14.97 ± 4.81	14.23 ± 1.51**	12.53 ± 2.89	14.42 ± 1.04***	20.00 ± 2.13*	13.37 ± 0.81**	20.76 ± 2.35***
DMSO [3]	15.45 ± 1.29	11.28 ± 3.37	5.87 ± 0.53	9.31 ± 1.60	4.74 ± 0.95	4.37 ± 0.29	5.72 ± 0.68	8.04 ± 1.00
Extract C								
5 µg/mL	11.34 ± 0.46*	11.02 ± 3.84	18.17 ± 4.79*	12.32 ± 2.49	9.85 ± 0.94	14.85 ± 2.64	13.54 ± 0.86**	16.47 ± 2.26*
10 µg/mL	11.32 ± 0.64*	10.90 ± 4.45	9.36 ± 1.03	11.78 ± 3.14	10.51 ± 0.41	17.81 ± 5.15*	14.12 ± 1.54***	17.05 ± 2.44*
20 µg/mL	11.68 ± 0.82*	11.64 ± 1.68	11.26 ± 0.63	11.53 ± 3.39	11.35 ± 1.20*	16.12 ± 1.66	15.55 ± 0.22***	14.77 ± 1.02*
DMSO [3]	7.78 ± 1.03	13.87 ± 1.59	5.51 ± 0.03	10.02 ± 1.25	7.25 ± 1.09	5.53 ± 0.74	6.57 ± 0.59	7.14 ± 1.40
Extract D								
0.5 mg/mL	42.49 ± 2.50***	26.64 ± 2.95*	75.04 ± 1.63***	84.22 ± 1.11***	18.44 ± 3.56**	22.99 ± 3.01*	39.52 ± 5.56***	46.50 ± 3.61***
1 mg/mL	43.85 ± 5.55***	39.78 ± 2.79***	55.17 ± 10.32**	74.27 ± 2.04***	18.31 ± 1.15**	20.53 ± 8.16	35.94 ± 0.43***	40.47 ± 1.88***
2 mg/mL	35.55 ± 3.25**	34.36 ± 5.79**	50.84 ± 3.37**	82.75 ± 1.45***	16.69 ± 1.76**	15.95 ± 1.83	14.52 ± 2.54	32.45 ± 0.41***
DMEM [2]	8.13 ± 1.94	9.00 ± 1.43	14.31 ± 1.13	8.57 ± 1.01	3.65 ± 1.02	3.81 ± 0.15	4.35 ± 0.59	4.73 ± 0.26
Positive controls:								
Valinomycin [4]	39.72 ± 5.46	54.91 ± 9.62	63.47 ± 6.53	73.19 ± 4.70				
Staurosporine [5]					7.55 ± 1.02	14.89 ± 1.63	29.64 ± 3.21	38.03 ± 5.59

[1] Early and late apoptosis were determined using the three highest concentrations which did not show cytotoxic effects in the LDH leakage test, starting by the top concentration (see Table 1). [2,3] Negative control for samples A and D, and B and C, respectively. [4,5] Positive control for early and late apoptosis, respectively. Statistical analysis: *(p<0.05), **(p<0.01), or ***(p<0.001) indicate significantly differences, as compared with control cells, one-way ANOVA followed by Dunnett's post hoc analysis.

Table S6. Effects of various ALEs concentrations on early (mitochondrial membrane depolarization; ΔΨm) and late (DNA fragmentation) apoptosis induction in RKO cells. Experimental groups comprised cells treated for 4, 8, 16 and 24 h. Results are expressed as percent of apoptotic cells, experimental sets are summarized as the mean (± SEM) of three independent experiments.

	**ΔΨm (Early apoptosis)**[1]				**DNA fragmentation (Late apoptosis)**[1]			
**ALEs**	**4 h**	**8 h**	**16 h**	**24 h**	**4 h**	**8 h**	**16 h**	**24 h**
Extract A								
1 mg/mL	34.81 ± 1.61***	32.89 ± 0.14	36.90 ± 0.50	56.72 ± 3.28***	23.65 ± 6.49	43.16 ± 2.29***	18.81 ± 2.72**	43.43 ± 0.33***
2 mg/mL	10.21 ± 4.34	61.39 ± 4.58**	3.67 ± 0.83	7.45 ± 0.57	44.12 ± 0.45***	34.91 ± 2.88***	47.94 ± 3.11***	62.14 ± 3.46***
4 mg/mL	1.96 ± 0.30	46.69 ± 8.50*	24.44 ± 11.04	39.70 ± 9.85*	60.45 ± 4.07***	51.91 ± 0.53***	66.32 ± 0.46***	10.28 ± 0.13
DMEM [2]	7.58 ± 1.91	23.80 ± 2.47	17.73 ± 2.84	13.53 ± 1.84	11.30 ± 3.33	10.10 ± 0.29	6.01 ± 0.92	4.90 ± 1.44
Extract D								
0.5 mg/mL	35.50 ± 4.51**	56.46 ± 1.46***	87.67 ± 5.48***	92.91 ± 2.18***	30.32 ± 2.87***	25.54 ± 1.48**	19.78 ± 0.10***	22.78 ± 3.25**
1 mg/mL	31.27 ± 4.56**	80.81 ± 2.31***	94.67 ± 1.84***	98.62 ± 0.04***	23.43 ± 0.77*	12.09 ± 2.52	17.43 ± 2.13***	15.08 ± 4.28
2 mg/mL	34.69 ± 2.62**	73.14 ± 5.59***	97.87 ± 0.59***	85.89 ± 2.26***	25.44 ± 1.91**	18.84 ± 4.49	15.30 ± 1.76**	11.58 ± 0.84
DMEM [2]	7.67 ± 1.72	22.75 ± 2.06	16.71 ± 2.25	12.98 ± 1.95	11.47 ± 2.31	10.05 ± 0.58	5.99 ± 1.03	4.87 ± 1.79
Positive controls:								
Valinomycin [3]	21.79 ± 8.56	33.88 ± 0.64	29.34 ± 8.13	33.88 ± 6.68				
Staurosporine [4]					39.78 ± 6.37	50.73 ± 1.55	71.48 ± 6.90	67.26 ± 1.81

[1] Early and late apoptosis were determined using the three highest concentrations which did not show cytotoxic effects in the LDH leakage test, starting by the top concentration (see Table 1). [2] Negative control. [3,4] Positive control for early and late apoptosis, respectively. Statistical analysis: *(*p*<0.05), **(*p*<0.01), or ***(*p*<0.001) indicate significantly differences, as compared with control cells, one-way ANOVA followed by Dunnett's post hoc analysis.

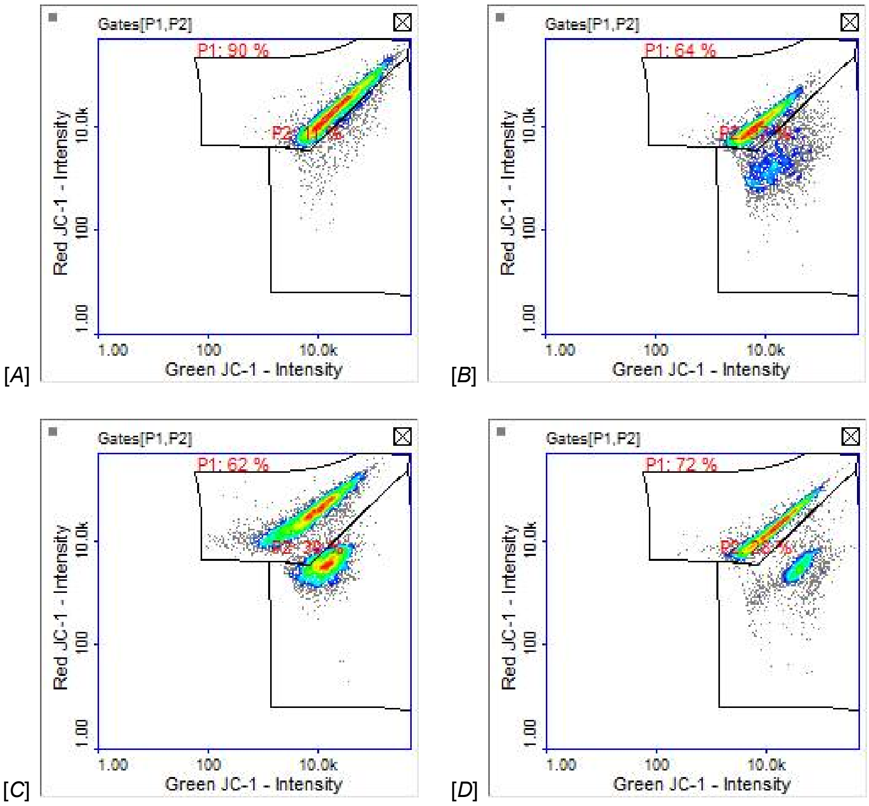

Figure S1. Determination of apoptosis (early events) by evaluation of mitochondrial membrane potential (ΔΨm). Green and red fluorescence of the viable cells displayed in scatterplots: [*A*] negative control; [*B*] positive control (0.5 µM valinomycin); [*C*], [*D*] artichoke leaf extracts A (1 mg/mL), and D (0.5 mg/mL), respectively.
